# Molecular typing of *Mycoplasma synoviae* in industrial and backyard poultry: a 14-year study in Italy

**DOI:** 10.1128/aem.01324-25

**Published:** 2026-03-12

**Authors:** Elisabetta Stefani, Ana Y. Morales-Arce, Giorgia Nai, Verdiana Righetti, Beatrice Colò, Davide Prataviera, Martina Giacomelli, Salvatore Catania

**Affiliations:** 1 Istituto Zooprofilattico Sperimentale delle Venezie, SCT1, WOAH Reference Laboratory for avian mycoplasmosis, Verona, Italy; Washington University in St. Louis, St. Louis, Missouri, USA

**Keywords:** backyard poultry farms, industrial poultry farms, MLST, *Mycoplasma synoviae*

## Abstract

The incidence of *Mycoplasma synoviae* (*M. synoviae*) in poultry farms is closely tied to intensive production systems, where high-density flocks and shared operational networks amplify transmission risks. Northern Italy is a densely populated poultry area (DPPA) of industrial and backyard poultry farms providing a unique setting to study *M. synoviae* dynamics, particularly given the integrated production model—where companies manage geographically dispersed farms with shared logistics. Using multi-locus sequence typing (MLST), we analyzed 227 *M. synoviae* Italian samples (181 industrial, 46 backyard) collected over 14 years (2010–2024) to assess transmission patterns and genetic diversity. Our results revealed high genetic diversity (99 sequence types, 95 novel). Transmission was linked to company networks, as identical strains circulated across farms under shared ownership, while backyard and industrial sectors showed minimal overlap. Phylogenetic analysis confirmed country-specific clustering. Moreover, population genetic analyses showed that Italy’s diversity was intermediate between the highly diverse USA and the more homogenized Chinese populations, with variability measures further suggesting distinct evolutionary pressures across genes and countries. Finally, this study challenges assumptions about backyard farms as reservoirs and shows that *M. synoviae* spread in Italy is primarily driven by industrial logistics. Our findings also highlight the value of MLST in tracing outbreaks at the company level, supporting its adoption for poultry pathogen surveillance. By providing the first long-term, system-wide perspective on *M. synoviae* epidemiology in a DPPA, this work offers critical insights for managing pathogen spread in integrated production networks worldwide.

**IMPORTANCE:**

This study provides valuable insights into the genetic diversity, spread, and evolutionary dynamics of *M. synoviae* in Italy. By analyzing genetic differences across isolates, we discovered two critical patterns: first, these bacteria tend to stay within national poultry production systems, forming country-specific genetic groups; second, within Italy, identical strains circulate within the same companies rather than by region. This suggests the pathogen spreads through poultry trade or shared farming practices rather than local environmental transmission. Surprisingly, we found no evidence of the bacteria moving between small backyard farms and large industrial operations despite concerns about such cross-transmission. The persistence of certain strains over time demonstrates the bacterium’s ability to survive even with strict biosecurity measures in industrial settings. Future studies should focus on the mechanisms that drive the persistence and spread of *M. synoviae* strains, as well as on the potential role of new transmission vectors and potential reservoirs.

## INTRODUCTION

The occurrence and persistence of poultry pathogens, such as mycoplasmas, are closely linked to intensive poultry production systems, which amplify the risk of exposure and transmission. In northern Italy, there is a densely populated poultry area (DPPA) where large concentrations of industrial poultry farms are found in a small geographic area along with backyards, making it an interesting case study for observing poultry pathogen dynamics. Chickens and turkeys are the most common avian farming species in this territory; though, guinea fowl, pheasants, partridges, ducks, and geese are also found. Furthermore, industrial poultry farming in DPPA is typically characterized by an integrated system in which all production phases are managed by few companies. These companies often operate multiple farms across different regions—sometimes separated by considerable distances—but share transportation, personnel, and fomites, all of which could contribute to pathogen spread. Furthermore, industrial systems rely on a limited number of commercial breeds, such as Ross 308 for broiler chickens and Hy–Line or Lohmann for laying hens, with specific breeds like B.U.T. 6 or Hybrid ConverterNOVO used for turkeys (personal communication). These production systems are characterized by the use of high-density flocks. In contrast, backyard farms are small-scale operations with low-density flocks (usually fewer than 50 birds), owned by private individuals. Backyard flocks are typically more diverse: multiple species and breeds often coexist, and the breeds used are different from those in industrial settings, reflecting a broader host-genetic background.

One of the relevant bacterial species in the intensive poultry industry causing considerable economic losses is *Mycoplasma synoviae* (*M. synoviae*). *M. synoviae* infection leads to airsacculitis and infectious synovitis in chickens and turkeys and is associated with EAA (Eggshell Apex Abnormality), consequently causing production losses in chicken layers (1–3). *M. synoviae* can be transmitted vertically and horizontally between individuals within the same flock or between flocks (4). Controlling *M. synoviae* dissemination involves biosecurity measures, vaccination, antibiotic treatments, and control programs using molecular markers, serological techniques, and typing of strains. However, these measures are commonly applied in the industrial sector but not in backyards (5). Therefore, the presence of infected backyards alongside industrial farms in the same territory could influence *M. synoviae* transmission between the two sectors (6). For all these reasons, the genetic identification of circulating field strains is a useful tool for tracking *M. synoviae* spread.

The main molecular identification technique for *M. synoviae* strains is multi-locus sequence typing (MLST), which can be used to infer the clonality and evolutionary relatedness of field strains (7). In particular, for *M. synoviae*, El-Gazzar and colleagues (8) developed a protocol using seven housekeeping genes (*adk*, *atpG*, *efp*, *gmk*, *nagC*, *ppa*, *recA*) covering around 3,959 bp in total; each allelic gene profile and their combinations define the sequence type (ST). Moreover, this methodology offers excellent accessibility through worldwide web databases, such as PubMLST (http://pubmlst.org/), which provides updated reporting and distribution of various sequence types (9). The free sharing of STs and strain information has enabled the identification of global distribution patterns as well as unique allelic combinations per country. Additionally, ST profiles are useful to estimate genetic diversity indexes which allow for population structure analyses, evolutionary patterns, and phylogenetic relationships. MLST is also widely recognized for its ease of use, scalability, and reliability, allowing for consistent implementation across various laboratories (10, 11). In contrast, while whole and core genome sequencing offer enhanced discriminatory power, they are often limited by higher costs and substantial computational requirements. Thus, MLST remains a practical and effective choice, providing sufficient discriminatory capabilities without the need for the extensive resources demanded by next-generation sequencing (NGS) techniques (12).

For the first time, we have applied MLST to track the spread of *M. synoviae* over extended periods in specific settings, such as companies, single entity farms, and backyards. While previous studies have used MLST to discriminate vaccine strains from field strains (13), identify predominant genotypes—including tracking antibiotic resistance genes and virulence factors (14)—and explore the phylogenetic origins of international strains (15–17), none have focused on long-term analysis of company-specific transmission dynamics. In our study, we hypothesized that transmission is influenced either by the proximity of farms or by shared ownership within the same company. To test this, we considered both factors in our analysis. Furthermore, we argue that Italian farms carry unique STs and, if shared, these STs are likely exchanged with European Union (EU) countries that trade products with Italy.

More specifically, we applied the MLST method (8) to type 181 industrial and 46 backyard field strains collected over fourteen years (2010–2024) across different Italian regions. The majority of our samples were constituted by isolates from chickens (∼78%) and turkeys (∼20%). This distribution reflects the structure of poultry farming in Italy, where production is heavily dominated by broilers (approximately 450 million birds per year), followed by laying hens (50 million) and turkeys (24 million). Moreover, the production cycle differs across species: broilers are typically raised for about 63 days before slaughter, whereas turkeys remain on farms for up to 6 months, potentially influencing exposure and transmission dynamics.

Our aim was to investigate *M. synoviae* outbreaks by identifying the STs over time and tracking their geographic dissemination in relation to farms belonging to the same companies, as well as between single-entity and backyard poultry farms. We examined the distribution of STs in infected flocks at the geographical (regional) level, as well as at industrial and backyard levels, and analyzed the genetic diversity and global context of Italian STs. Additionally, we analyzed the genetic variation and evolutionary relationships of Italian strains, comparing them with data derived from different countries obtained from PubMLST.

Our results demonstrated that STs would be more likely shared between the same companies rather than by region. In addition, we found that the presence of backyards does not affect industrial poultry farms or vice versa, except in exceptional cases. Additionally, we show that the MLST genes are useful to distinguish between continents and countries. Finally, this is the first study applying the MLST approach in Italy to track *M. synoviae* diffusion in a highly populated farming area, including backyard contexts along with companies over a long period of time.

## MATERIALS AND METHODS

### Sample collection and study area

We analyzed 227 Italian samples of *Mycoplasma synoviae* (*M. synoviae*) collected from 2010 to June 2024 from chickens (*n* = 176), turkeys (*n* = 44), guinea fowls (*n* = 4), partridge (*n* = 1), goose (*n* = 1), and pheasant (*n* = 1) (Table S1). The study was performed on residual tracheal swab specimens collected from poultry exclusively within routine diagnostic and surveillance activities. No animals were sampled specifically for research purposes and no additional procedures were carried out for the aims of this study. In accordance with Italian legislation (D.Lgs. 26/2014 implementing Directive 2010/63/EU), no ethical approval under the animal experimentation framework was required. Owner informed consent was not applicable beyond routine diagnostic submission and samples were analyzed in anonymized form. More than 80% of collections (*n* = 181) were from industrial poultry sources; the remaining were from backyard farms (*n* = 46). Samples were collected from different farming areas, especially in the northern regions: Veneto (*n* = 92), Emilia Romagna (*n* = 69), Lombardia (*n* = 15), Friuli Venezia Giulia (*n* = 5), Trentino Alto Adige (*n* = 4), and Piemonte (*n* = 3). Other central and southern regions were less represented: Abruzzo (*n* = 12), Marche (*n* = 6), Molise (*n* = 3), Sicilia (*n* = 9), Toscana (*n* = 4), and Umbria (*n* = 2). About 90% of backyard farm samples (*n* = 41) were collected in the Veneto region from 2010 to 2021. Because the Veneto region contained most backyard poultry samples, it was selected for a focused comparison of *M. synoviae* diffusion within a restricted geographic area to assess possible epidemiological connections between backyard and industrial sectors. Finally, the industrially sourced samples mostly came from three companies (labeled A, B, and C), consisting of various farms located in different regions. We collected 38 samples from company A, 53 from B, and 37 from C. The remaining 53 samples came from different minor industrial poultry farms (named as D). The regions included in this study largely overlap with the designated poultry production area (DPPA) of northern Italy, as defined by national and EFSA criteria (18). This area encompasses most of Italy’s industrial poultry production, with more than 3,000 farms and approximately 95 million bird places, accounting for nearly 70% of the country’s intensively reared poultry (19). Farm densities in the DPPA exceed 0.52 farms/km^2^, with bird densities averaging around 10,000 birds/km^2^ and reaching up to 70,000 birds/km^2^ in some northern provinces. As our sampling was concentrated in these high-density regions, the dataset represents a cross-section of *M. synoviae* diversity within Italy’s main poultry production zone (18, 19).

### *Mycoplasma synoviae* isolation

*M. synoviae* was isolated according to the WOAH Terrestrial Manual (Chapter 3.3.5, 2021). Briefly, tracheal swabs were collected from symptomatic birds or from flocks that tested positive in molecular screening, regardless of the presence or absence of clinical signs in individual animals, and placed in 1 mL of transport medium (Avian Mycoplasma Experience, Mycoplasma Experience Ltd, UK) and maintained at 4°C until arrival at the laboratory. Each sample was filtered using a 0.4 μm filter and subsequently inoculated into 2 mL of medium and incubated at 37°C under 5% CO2 for 21 days (WOAH Terrestrial Manual, Chapter 3.3.5, 2021). During this period, the broths were checked daily; if a change in color or turbidity was recorded, they were inoculated onto an agar plate of Avian Mycoplasma Experience (METM, Mycoplasma Experience, Reigate, UK) and incubated under the same conditions. All plates were then checked daily for the presence of any “fried-egg” colony for up to 7 days. Samples were considered negative when no changes were observed in either broth or agar.

### Nucleic acid extraction and identification

DNA was extracted from suspect broths using the Maxwell DNA LEV Blood Kit and the Maxwell 16 Instrument (Promega, Milan, Italy) following the manufacturer’s instructions. The extracted DNA underwent 16S rDNA PCR and denaturing gradient gel electrophoresis (DGGE) as described in the literature (20), for Mycoplasma species identification. In cases where isolation was not possible, tracheal swabs diluted in PBS/D were processed using the MagMAX Pathogen DNA/RNA kit (Applied Biosystems) following the manufacturer’s instructions. The DNA was then analyzed by Real-Time PCR to verify the presence of *M. synoviae* following WOAH Terrestrial manual guidelines (21).

### *Mycoplasma synoviae* MLST

The DNA of samples identified as *M. synoviae* was amplified using primers of the seven housekeeping genes *adk*, *atpG*, *efp*, *gmk*, *nagC*, *ppa*, and *recA* listed in the PubMLST database (9). Each PCR was performed in a 20 μL volume containing 10 μL Sybr Fast Universal Master Mix (2×) (Merck Life Science, Milan, Italy), gene-specific primers at 0.1 μM, and 1.5 μL of DNA. PCR cycling included a hot start at 95°C for 3 min, followed by 35 cycles of denaturation (94°C, 1 min), annealing (54°C, 30 s), and elongation (72°C, 90 s). The BioRad CFX96 Thermal cycler (BioRad, Milan, Italy) was used for amplification, and the fluorescence in the FAM/Sybr channel was acquired at the end of the elongation step. PCR products were subjected to Sanger sequencing using the same primers. New alleles and STs were submitted to the PubMLST database.

### Simpson’s index of diversity

The Simpson’s index of diversity (SID) gives the probability that two unrelated isolates sampled randomly from the population will be assigned to different sequence types. The SID was calculated according to the formula of Hunter and Gaston (1988) (22). The formula is as follows:


(1) $$D=1-\frac{1}{N(N-1)}\sum_{i=1}^{s}n_{i}(n_{i}(n_{i}-1)$$

where *D* is the Hunter–Gaston discriminatory index (HGDI), *N* is the total number of isolates in the dataset, *s* is the total number of types (e.g., sequence types, alleles, or profiles), and *n_i_* is the number of isolates belonging to the *i*th type.

The SID value ranges from 0 to 1, with 0 indicating no diversity and 1 representing infinite diversity.

### Minimum spanning tree analysis

Relationships among strains were evaluated using minimum spanning tree (MST) analysis generated by Bionumerics software v7.6 (Applied Math, Sint-Martens-Latem, Belgium). An MST is used to visualize the genetic relationships between different STs based on the number of allele differences between them. Strains were grouped into clonal complexes, in which every genotype shares at least 6 out of 7 loci with at least one other member of the group. The ST numbers were enlarged using Adobe Illustrator for better visual presentation.

### Phylogenetic analyses

The allele sequences of each housekeeping gene and the concatenated profile for each ST were downloaded from PubMLST in FASTA format. Our analysis included 220 sequences, combining internal metadata from our laboratory with PubMLST allele profiles globally reported up to June 2024. Sequences exceeding the expected concatenated gene size—typically due to insertions or deletions resulting from sequencing errors—were excluded, resulting in a final dataset of 3,959 positions.

Phylogenetic relationships among STs were then analyzed using multiple tools: MEGA v7.0.26 (23, 24) and iTOL v6.9 (25). The evolutionary history was inferred by using the Maximum Likelihood method based on the Tamura–Nei model (26). The tree with the highest log likelihood (−12,589.22) is shown. Initial tree(s) for the heuristic search were obtained automatically by applying Neighbor-Join and BioNJ algorithms to a matrix of pairwise distances estimated using the Maximum Composite Likelihood (MCL) approach and then selecting the topology with superior log likelihood value. The heterogeneity of substitution rates among sites was modeled with a discrete Gamma distribution (five categories; shape parameter *α* = 0.1198), which assumes that substitution rates vary across sites according to a Gamma distribution, allowing some genomic positions to evolve more slowly or more rapidly than others due to functional or structural constraints. The rate variation model allowed for some sites to be evolutionarily invariable ([+I], 46.31% sites). Codon positions included were 1st+2nd+3rd+Noncoding. All positions containing gaps and missing data were eliminated. Phylogenetic inference was performed in MEGA7 (Molecular Evolutionary Genetics Analysis v7), a widely used desktop package for sequence alignment, model selection, and phylogenetic reconstruction. In MEGA7, we specified the Tamura–Nei model with +G+I as above, obtained NJ/BioNJ starting trees, optimized model parameters and the ML topology, and exported the final tree for visualization and annotation in iTOL v6.9 (23).

### Genetic diversity indices

Genetic diversity analyses were conducted on samples from Italy and compared to those from China and the United States. We included only these two countries because they had the highest number of isolates available in PubMLST. The Chinese and U.S. sequences were retrieved directly from the public MLST database, which compiles previously published data from multiple studies. Metadata for these isolates are generally limited to the country and year of isolation, and details such as precise farm locations or husbandry systems are not consistently reported. Genetic diversity metrics were calculated using the Arlequin software (27), including the number of haplotypes (*h*), nucleotide diversity (π)—representing the mean number of nucleotide differences per site between two randomly chosen DNA sequences—and the number of segregating sites (*S*) per population. We also calculated Tajima’s *D*, a statistic that compares different estimates of genetic variation to test for deviations from neutrality; values significantly different from zero may indicate demographic events such as population expansion, bottlenecks, or selection. Finally, we estimated the fixation index (*F*_ST_), which measures genetic differentiation between populations. *F*_ST_ values range from 0 to 1, with higher values (e.g., >0.25) indicating strong genetic differentiation and limited gene flow between populations, while lower values suggest greater genetic similarity.

## RESULTS

### MLST analysis of Italian strains over time and across regions

In this study, we analyzed 227 Italian *M. synoviae* isolates collected from 2010 to 2024 from three major companies (A, B, and C), as well as from smaller industrial poultry farms (*D*) and backyard settings across various regions, using MLST. The isolates were classified into 99 STs, yielding a SID value of 0.96, indicating a high degree of diversity. We identified 95 new STs, attributed to either novel allelic sequences or new combinations (Table S1). All isolates grouped into 12 clonal complexes (CCs), with 41 isolates remaining as singletons.

**Fig 1:**
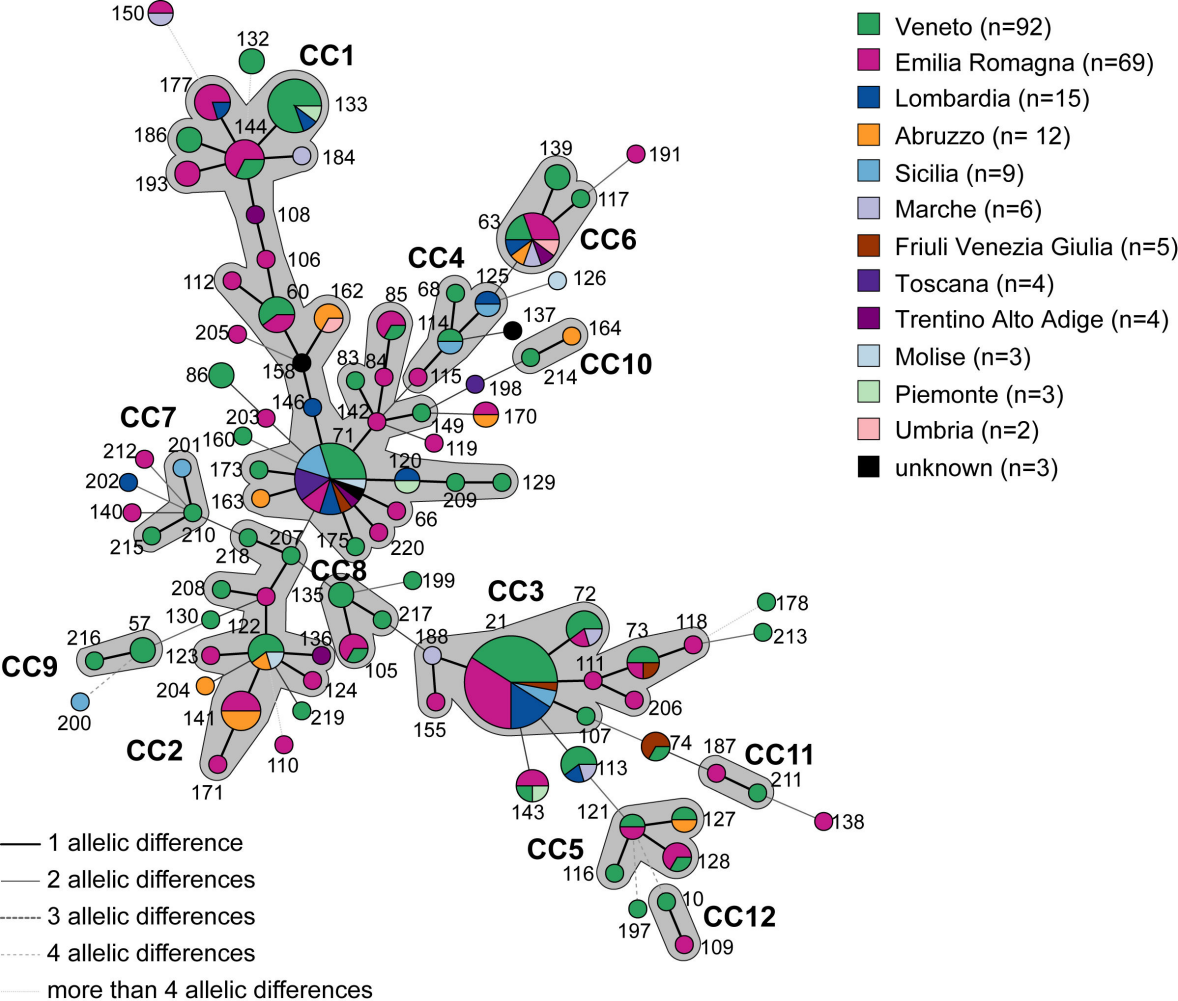
The minimum spanning tree indicates the relationship between STs of Italian *M. synoviae* samples highlighting the regions where samples were collected. Each circle corresponds to a sequence type (ST). Number near circles indicates ST number. The size of the circle represents numbers of isolates with same STs. Lines between STs indicate inferred phylogenetic relationships. The style and thickness of the line represent the number of allelic mismatches between the STs (thick line = 1, thin line = 2, thick dashed = 3, thin dashed = 4, and thin dotted = more than 4). Gray colored zones that surround STs indicate the different CC (clonal complex). The color of the circle indicates the Italian region of farm where strains were isolated.

**Fig 2:**
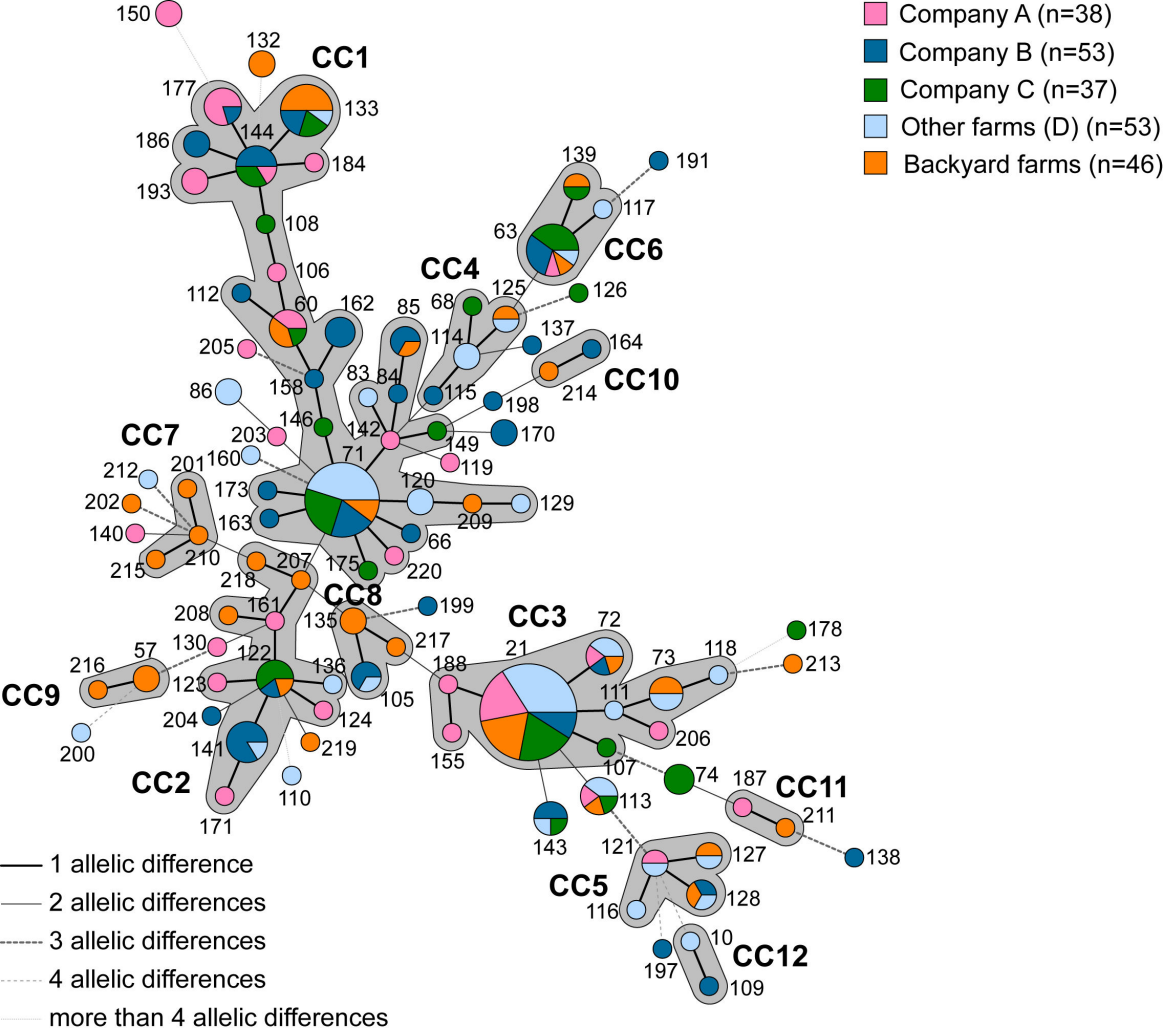
The minimum spanning tree indicates the relationship between STs of Italian *M. synoviae* samples highlighting the companies or farms where samples were collected. Each circle corresponds to a sequence type (ST). Number near circles indicates ST number. Size of the circle represents numbers of isolates with same STs. Lines between STs indicate inferred phylogenetic relationships. The style and thickness of the line represent the number of allelic mismatches between the STs (thick line = 1, thin line = 2, thick dashed = 3, thin dashed = 4, and thin dotted = more than 4). Gray colored zones that surround STs indicate the different CC (clonal complex). The color of the circle indicates the company or farm where strains were isolated.

Most isolates clustered into the same clonal complexes regardless of sector or regional origin (Fig. 1 and 2). For example, within CC1, STs 60, 71, 85, and 133 were shared by several backyard and industrial isolates from different regions. However, some isolates from the same sector also clustered within specific CCs: in CC7 and CC9, only backyard samples were found, while CC12 contained only industrial samples (Fig. 2).

**Fig 3:**
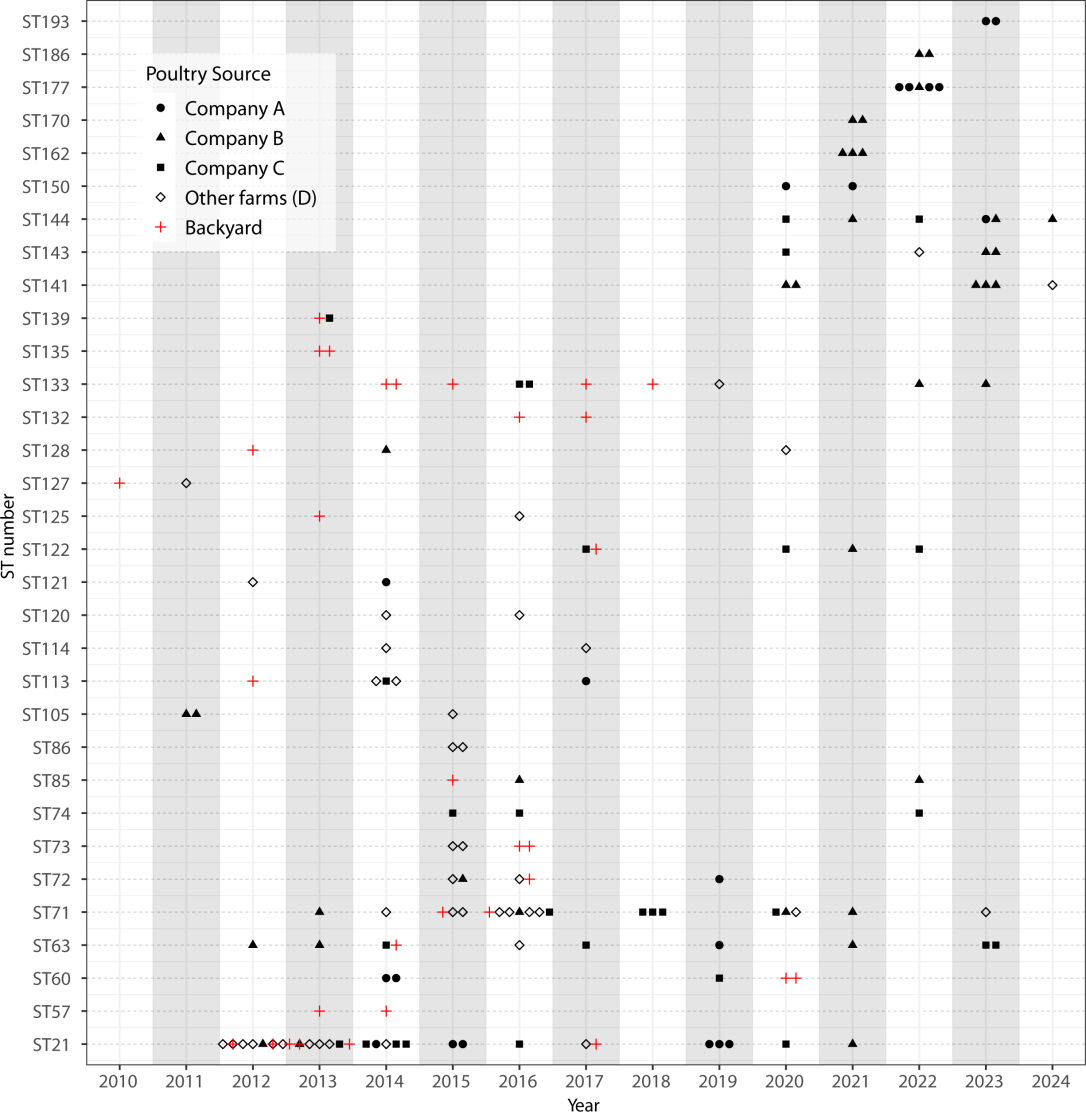
Yearly distribution of STs from industrial and backyard poultry farms between 2010 and 2024. Only STs repeatedly observed over time are shown, grouped by poultry source: commercial producers (Companies A–C), other industrial farms and backyard flocks.

The most frequently encountered STs over the years were ST21 (*n* = 32), ST71 (*n* = 20), ST63 (*n* = 10), and ST133 (*n* = 10) (Table S1), with each shared across both sectors. ST21 was primarily collected during the 2012–2014 period but progressively disappeared and was no longer isolated after 2021, while ST71 was predominantly found from 2015 to 2016 and subsequently persisted only in the industrial sector until 2023 ( Fig. 3).

Although fewer in number, ST71 and ST63 isolates were detected over a longer period, with ST71 also being collected from a greater number of regions than ST21 (Fig. 1). Furthermore, ST133 initially showed a preference for rural settings before becoming established in the industrial circuit (Fig. 3).

Most STs (*n* = 67) were represented by a single isolate (Fig. S1) over a span of 14 years. Despite some of these strains being collected many years ago, they have not reappeared (e.g., STs 116, 138, and 218). Conversely, 32 STs were shared among multiple isolates, and some STs appeared only in specific sectors, such as STs 114 and 121 in industrial, and STs 57 and 132 in backyard farms (Fig. 3).

Finally, we observed that some STs were present in multiple host species (e.g., ST21, ST60, and ST85), suggesting that certain STs can circulate among diverse hosts (Table S1).

### *M. synoviae* spread in Italian companies over time and across regions

MLST analysis of strains collected from poultry farms over time is a crucial tool for tracking the spread of *M. synoviae* within companies. In Italy, three major companies often operate multiple farms located in different regions, sometimes many kilometers apart. The MLST analysis of samples from these three companies A, B, and C across various farms and regions showed that the same STs were sometimes found within each company. For example, ST21 was isolated in 2019 from three different farms belonging to company A in Emilia-Romagna, ST186 in 2022 from two farms of company B, and ST133 in 2016 from two farms of company C in other northern regions (Fig. 1 to 3 and Table S1). Conversely, identical STs were also found within the same company in farms located far apart. For instance, ST162 was isolated in 2021 from three farms owned by Company B across two regions far from the DPPA. Similarly, ST71 was found in 2018 in Company C from three farms in the north of Italy (Fig. 1 to 3 and Table S1). The same ST can be re-observed across different years, sometimes consecutively, either within the same company or across different companies, suggesting strain persistence in the industrial sector (Fig. 3). Notable examples include STs 21, 71, 113, 122, 143, and 144, which were identified in different years and companies, as well as STs 74, 141, and 150, which were detected in the same companies over multiple years.

### Backyard poultry farms collected in the Veneto region

**Fig 4:**
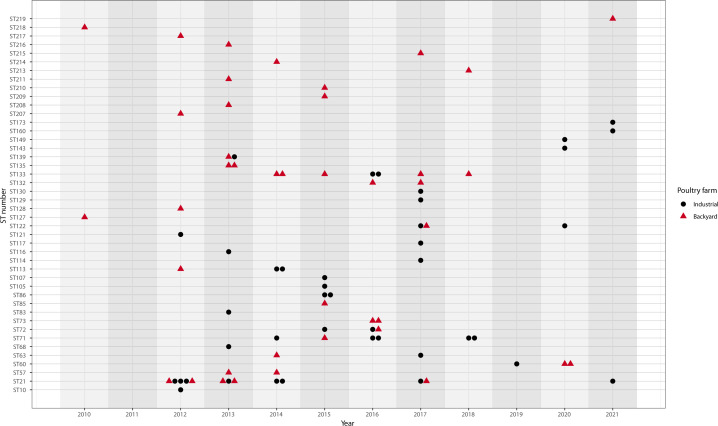
Distribution over time of STs from isolates collected in the Veneto region between 2012–2021. STs are grouped by source—industrial or backyard farms. The figure highlights persistence and sector-specific recurrence of certain STs across the study period.

We identified 44 STs, 19 STs were exclusive to backyard samples, 16 were unique to industrial samples, and 9 STs were shared by both sectors (Fig. 4). In some instances, such as ST21, ST72, ST122, and ST139, industrial and backyard farms carried the same ST within the same year, suggesting persistence of the same *M. synoviae* strain in the region. However, all the other STs were not shared between sectors during the same year, indicating limited interaction.

### Phylogenetic analysis

**Fig 5:**
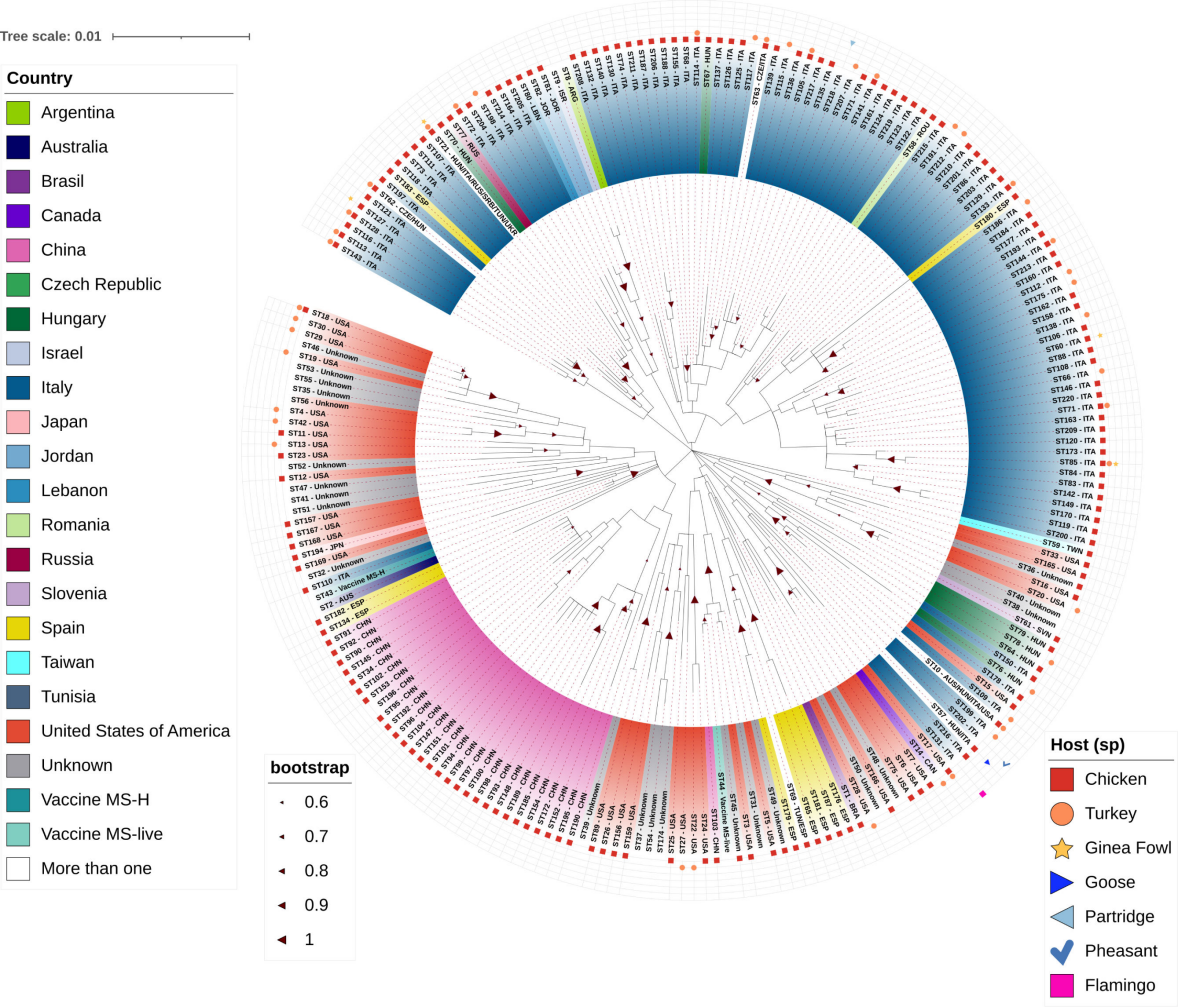
Phylogenetic tree of *Mycoplasma synoviae* based on 220 concatenated sequences of isolates recorded in PubMLST. Sequence types (STs) are labeled with their country of origin (e.g., Argentina, Italy, USA) and host species (e.g., chicken, turkey, guinea fowl). The tree includes all STs available in the PubMLST database to provide a global phylogenetic context. Italian STs are highlighted to emphasize their distribution and clustering among worldwide isolates. Bootstrap support values (0.6–1) are shown on branches, and the scale bar represents 0.01 substitutions per site. The vaccine strains MS-H and MS-live are included for comparison.

To infer evolutionary relationships among *M. synoviae* isolates, a Maximum Likelihood tree was constructed based on the concatenated MLST sequences recorded on PubMLST (Fig. 5).

We observed a large number of globally distributed STs. Most STs clustered by country, indicating nation-specific trends, with some exceptions. For instance, ST21 (Hungary, Italy, Russia, Serbia, Tunisia, Ukraine) was the most widely shared, followed by ST10 (Australia, Hungary, Italy, USA) and ST57 (Hungary and Italy). Interestingly, Italian STs clustered with other European isolates and those from the Middle East (Lebanon, Israel, Jordan). Finally, STs of different host species did not form separate clusters, indicating they do not contribute to the tree’s structure (Fig. 5).

### Genetic analysis

For genetic analysis, we focused on isolates from Italy, China, and the USA, as these countries represented those with the highest number of isolates uploaded to PubMLST. Italian samples harbored novel alleles in all loci while Chinese strains had lower number of unique alleles, in particular in the *efp* (2), *atpG* (2), and *ppa* (2) (Table 1). Generally, the USA and Italy showed higher private (population-specific) allele counts (e.g., *nagC*: Italy = 20, USA = 17; *atpG*: Italy = 11, USA = 16; *recA*: Italy = 12, USA = 10) (Fig. S2B, G, and E, respectively). Furthermore, the *nagC* gene exhibited the highest number of segregating sites (*S*) overall, with Italy and the USA both at *S* = 32, followed by China (*S* = 28). In contrast, the *efp* gene displayed the lowest allele counts and *S* across all populations. For concatenated genes, Italy had the highest number of unique STs (*n* = 98), while China had the fewest (*n* = 30). Additionally, *S* in concatenated sequences followed a distinct trend: the USA ranked highest (*S* = 137), Italy intermediate (*S* = 116) and China lowest (*S* = 79). Mean pairwise differences (π) also followed this order: USA (π = 24.09), Italy (π = 14.10), China (π = 8.85) (Table 1). These differences likely reflect distinct population histories: the USA’s high diversity may result from its long sampling period (1955–2021) and stable population, whereas China’s low diversity could indicate a recent bottleneck or founder effect.

**TABLE 1:** Genetic variability analysis of MLST data covering 7 gene loci in strains from Italy (2010–2024), China (2016–2024), and the United States (1955–2021)^*a*^

Country	Gene	Gene product	Length (bp)	Haplotypes (*h*)	Unique alleles	Seg. sites (*S*)	Pairwise diff. (*π*)	Tajima's D (*D*)	*F*_ST_
Italy	adk	Adenylate kinase	498	15	10	16	1.72	−0.89	0.46
China				6	4	11	1.39	−0.65	
USA				15	9	16	2.32	−0.92	
Italy	atpG	ATP synthase gamma chain	669	13	11	18	3.20	0.17	0.55
China				3	2	8	0.14	−2.00	
USA				19	16	25	4.81	−0.26	
Italy	efp	Elongation factor P	450	7	4	9	1.71	0.32	0.56
China				5	2	5	0.41	−1.01	
USA				10	5	7	1.79	0.54	
Italy	gmk	Guanylate kinase	470	16	11	16	0.99	−1.59	0.33
China				6	3	8	0.17	−1.95	
USA				12	6	16	2.92	−0.39	
Italy	nagC	N-acetylmannosamine kinase	708	24	20	32	2.99	−1.23	0.31
China				15	11	28	4.01	−0.48	
USA				21	17	32	5.01	−0.82	
Italy	ppa	Inorganic pyrophosphatase	453	8	5	10	2.38	0.99	0.57
China				3	2	6	0.30	−1.46	
USA				16	12	18	3.13	−0.52	
Italy	recA	Recombination protein	711	14	12	15	1.08	−1.42	0.50
China				8	3	13	2.41	0.18	
USA				15	10	23	4.08	−0.49	
				No. of unique STs					
Italy	Concatenated	Not applicable	3959	99	98	116	14.10	−0.83	
China				30	30	79	8.85	−1.08	
USA				37	36	137	24.09	−0.57	
All countries				165	235		20.90	−1.20	0.47

^*a*^ Sample sizes: Italy = 227, China = 181, USA = 65.

Tajima’s *D* values—a statistic comparing the number of segregating sites with the average pairwise differences to detect departures from neutrality—for concatenated genes were negative in all countries, indicating an excess of rare alleles and potential population expansion or purifying selection. China had the strongest deviation (*D* = −1.08), followed by Italy (*D* = −0.83) and the USA (*D* = −0.57). Gene-specific Tajima’s *D* values varied; notably, the positive Tajima’s *D* value (0.99) for the *ppa* gene suggests an excess of intermediate-frequency polymorphisms, which may be indicative of balancing selection, a recent population bottleneck, or a combination of both. Further analysis would be needed to distinguish between these evolutionary forces, but the result clearly points to unique evolutionary dynamics acting on this gene compared to the others.

Finally, *F*_ST_ values—which quantify genetic differentiation among populations based on allele frequency variation—revealed the highest population differentiation in *ppa* (0.57), *efp* (0.56), and *atpG* (0.55). For concatenated genes, *F*_ST_ was 0.47, supporting a moderate to high population structure—that is, genetic subdivision among isolates from different geographic regions, reflecting limited gene flow between them. These results confirm the utility of the seven genes in distinguishing the three populations and suggest that genetic diversity is largely shaped by differences among populations rather than within them.

## DISCUSSION

This study represents the first attempt to assess the genetic diversity of *M. synoviae* within industrial farms and between industrial and backyard poultry farms in Italy. Using MLST as an epidemiological tool, we analyzed *M. synoviae* isolates to track the pathogen’s presence over a 14-year period. We also investigated how persistence shaped the spread of the pathogen within industrial farms and between industrial and backyard settings. Furthermore, we explored whether the continued presence of particular strains was influenced by their origin (i.e., specific farms or companies). Finally, we compared the phylogenetic relationships between Italian and global strains, providing new insights into the pathogen’s dissemination.

Our findings reveal a high level of *M. synoviae* genetic diversity, with 99 distinct STs, 95 of which were found exclusively in Italy (Fig. 5). Notably, this level of variation was observed despite sampling primarily from a single host species, a limited number of producers (most belonging to the three companies: A, B, and C) and a single country of origin. Consistent with this variability, we identified 95 novel allelic sequences or new combinations of existing alleles, highlighting the ongoing evolution of *M. synoviae* and its capacity to generate new strains. Our results align with previous studies that underscore the genetic variability of *Mycoplasma* species (28). Similar diversity has been reported across different countries and host species, including *M. hominis*, *M. capricolum* subsp. *capripneumoniae*, *M. gallisepticum*, *M. hyorhinis*, *M. iowae*, and *M. agalactiae* (17, 29–33). This diversity is likely driven by the inherent genetic plasticity of *Mycoplasma* species, which enables them to generate new genetic combinations through mutation, recombination, and selection (34, 35).

The high number of STs isolated only once (67 out of 99) (Fig. S1) could be the result of biosecurity measures such as *M. synoviae* vaccinations (implemented in Italy since 2012) and/or antibiotic use, potentially restricting strain transmission. In parallel, the sporadic detection of certain STs might reflect strain-specific differences in pathogenicity, particularly due to their tropism for distinct tissues. For example, oviduct-tropic strains could achieve broader spread via vertical transmission, such as *Mycoplasma iowae* (36) while tracheal-tropic strains might persist only in localized infections, favoring horizontal transmission. Mycoplasma species capable of both kinds of transmission and producing biofilm (37) might persist longer in time.

Furthermore, our samples were grouped into 12 CCs, across different geographic locations (Fig. 1) and industrial sectors (Fig. 2). Regardless of their geographic origin, samples belonging to the same CC often displayed different regions of provenance, suggesting no clear correlation between *M. synoviae* strains and their region of isolation. Furthermore, the more frequently an ST is found, the more regions it tends to encompass. In contrast, isolates from major poultry companies (Fig. 2) revealed identical STs circulating among farms separated by considerable distances, as exemplified by ST170, ST150, and ST162, which were detected across multiple farms within the same company despite their wide geographic dispersion. Additionally, a temporal analysis of ST recurrence revealed that certain outbreaks, both within the same year and across multiple years within the same company, were associated with the same STs (e.g., ST162 and ST141, respectively; Fig. 3). Despite biosecurity measures, this sharing of STs between companies shows that *M. synoviae* transmission is primarily driven by the logistics of the integrated production system, facilitating the spread of identical STs across farms rather than being influenced by geographic proximity. Other typing studies have similarly found that geography was not significantly related to genotype in *M. synoviae* (38). These findings align with previous observations in other poultry pathogens, such as infectious bronchitis virus (IBV), where transmission was linked to shared infrastructure (e.g., roads, personnel, and services) rather than environmental factors (39). Further research has shown that *M. synoviae* can produce biofilms on abiotic surfaces, enhancing survival in harsh conditions and facilitating long-distance spread (37). In addition, stable host populations (e.g., same breed and age) and high poultry densities create favorable conditions for persistence and transmission. The effect of vertical transmission cannot be excluded, as the companies included in this study have economic relationships involving the sale of hatching eggs.

Moreover, previous hypotheses have suggested that backyard poultry flocks can act as reservoirs or amplifiers for respiratory diseases (39–42). However, our data indicate that in the Veneto region, transmission between the two sectors is limited (Fig. 4). In fact, only 9 STs were shared between the two sectors. Among these, ST21 was the most widespread, while ST139 appeared exclusively in 2013. ST122 was first detected in both sectors in 2017 but subsequently persisted only in industrial farms. ST60, ST63, ST71, and ST72 initially emerged in the industrial sector (outside Veneto) and appeared transiently in backyard flocks before re-establishing in industrial systems, suggesting sporadic spillover from commercial to backyard production. An exceptional case, ST133, was first identified in rural flocks before stabilizing in industrial systems, implying rare cross-contamination from backyard to industrial sectors (Fig. 3 and 4). Nevertheless, backyard flocks are located within the high-density poultry production areas (DPPA) of the Veneto region, yet they are not subject to mandatory requirements. The fact that many STs were exclusive to one sector and did not overlap in the same year implies that these two types of poultry farming largely function as independent reservoirs.

The phylogenetic tree (Fig. 4) revealed that Italian *M. synoviae* isolates exhibited clear geographical specificity, as previously observed in MLST-based phylogenies (15, 16). However, we observed that some isolates from other European countries, as well as from the Middle East—particularly Lebanon, Israel, and Jordan—clustered with Italian strains. This pattern of global sharing of certain STs highlights the potential for international transmission of *M. synoviae* through poultry and product trade. For instance, ST21 was identified in Italy, Hungary, Serbia, Tunisia, Ukraine, and Russia, suggesting possible commercial exchanges. In another case, the ST10 was first reported in 1997 in the USA and continued to be found in different countries until 2016 such as Hungary and Austria and also found in Italy once in 2012 (Table S1). As previous studies have suggested (43), intercontinental exchanges could be due to the migration of wild birds carrying mycoplasmas. However, more studies are necessary to confirm and clarify the role of migratory birds in this transmission route. Moreover, the possibility of commercial exchange as a contributing factor cannot be excluded and should be considered in future investigations.

To further elaborate, the most represented countries in PubMLST are the USA, China, and Italy, which clustered separately, as evidenced by our phylogenetic analysis. Italy had the highest number of unique STs (98), compared to China (30), suggesting different evolutionary pressures. China showed a lower number of alleles in all genes compared to Italy and the USA. While Zhang et al. (15) reported that the least variable genes were *efp*, *gmk*, and *ppa*, Wei et al. (16) identified *ppa* and *atpG* as least variable in China. Our results confirm *atpG*, *efp,* and *ppa* as the least variable genes for China, reflecting a combination of previous findings. In Italy, variability was lowest in *efp* and *ppa*, but differently from China, not in *gmk* (Fig. S2-C, D and F). Genetic diversity (π) varied between countries: Italy (π = 14.10) showed intermediate diversity, possibly reflecting past population dynamics. The USA had a high value (π = 24.09), possibly due to long-standing endemic diversity or multiple introductions, while China’s low value (π = 8.85) suggests a more clonal population structure, though broader sampling would be needed to verify these patterns beyond the currently available PubMLST data. This country-specific variation likely reflects differences in environmental pressures, farming practices, breed genetics, and poultry movement networks.

Further, Tajima’s *D* values revealed distinct evolutionary pressures acting on *M. synoviae* populations across the three countries. All countries showed negative values (Italy: −0.83; USA: −0.57; China: −1.08), suggesting either recent population expansion—introducing an excess of rare alleles—or pervasive purifying selection. These patterns align with previous findings of predominant purifying selection in *M. synoviae* housekeeping genes (8). China’s strongly negative value (−1.08) supports the hypothesis of a recent expansion, possibly following a founder introduction, rather than a simple bottleneck alone, consistent with the proposed late introduction of *M. synoviae* to China (15). In turn, Asia’s rapid growth in poultry production over the past decade may have facilitated this expansion (44). In contrast, Italy’s more moderate negative value (−0.83) could be consistent with a longer history of purifying selection, with fewer recent demographic shifts which could be consistent with a combination of factors in the integrated Italian system, such as breed types, farming systems, biosecurity measures, and poultry density; nonetheless, population substructure across farms may also contribute to this signal. Our analyses suggest the Italian *M. synoviae* population may be genetically distinct from those of China and the USA, indicating divergent evolutionary trajectories shaped by local farming practices and host–pathogen dynamics.

Additionally, among the seven housekeeping genes, two*—nagC* and *ppa*—have dual metabolic roles. *nagC* participates in sialic acid catabolism and host adaptation (45), while *ppa* is involved in energy homeostasis during nutrient stress (46). These two loci are therefore likely subject to niche-specific selective pressures, as reflected in their diversity metrics. *nagC* showed high variation (32 segregating sites in both Italy and USA; Italy, π = 2.99, and USA, π = 5.01). Meanwhile, *ppa* shows the strongest population differentiation (*F*_ST_=0.57) and exceptionally divergent Tajima’s *D* values between the three countries, ranging from −1.46 (China) indicating purifying selection to 0.99 (Italy) suggesting balancing selection. In comparison, strict housekeeping genes, like *gmk* and *adk*, show lower diversity and consistently negative Tajima’s *D* suggesting predominant purifying selection. Their low diversity and negative Tajima’s *D* values may reflect functional constraints more than simple demographic history. For a clearer separation of demographic versus selective signals, inclusion of additional putatively neutral markers (e.g. 16S-23S spacer) might be recommended.

In conclusion, this study shows that industrial production networks predominantly drive *M. synoviae* transmission in Italy, rather than geographic proximity. High genetic diversity reflects a balance between evolutionary processes (e.g., purifying and balancing selection) and farm–level biosecurity practices, with only sporadic spillover from backyard flocks into integrated operations. These findings underscore the need for surveillance programs tailored to vertically integrated poultry systems, often referred to as DPPA, especially as global demand for poultry meat continues to drive the expansion of large–scale, high–density production models. Targeted monitoring within these networks will be crucial for early detection and control of *M. synoviae* outbreaks, but also to reduce its impact on production efficiency and promote more sustainable practices through improved disease management, reduced dependence on antibiotics (47) and, consequently, reduced risk of resistance development.

## Data Availability

All data supporting the findings of this study are available within the article and its supplemental material.

## SUPPLEMENTAL MATERIAL

Supplemental figures aem.01324-25-s0001.pdf Fig. S1 and S2.

Graphical abstract aem.01324-25-s0002.tif Visual depiction of the study.

Table S1 aem.01324-25-s0003.xlsx Background information of 227 *Mycoplasma synoviae* strains and summarized results of MLST.

ASM does not own the copyrights to Supplemental Material that may be linked to, or accessed through, an article. The authors have granted ASM a non-exclusive, world-wide license to publish the Supplemental Material files. Please contact the corresponding author directly for reuse.
