# Supplementary material for: Molecular typing of Mycoplasma synoviae in industrial and backyard poultry: a 14-year study in Italy
Source: Appl Environ Microbiol. 2026 Mar 12;92(4):e01324-25. doi: 10.1128/aem.01324-25 (PMC13101485; doi:10.1128/aem.01324-25)

Figure 1S. Backyard and industrial poultry samples isolated only once between 2010 to 2024

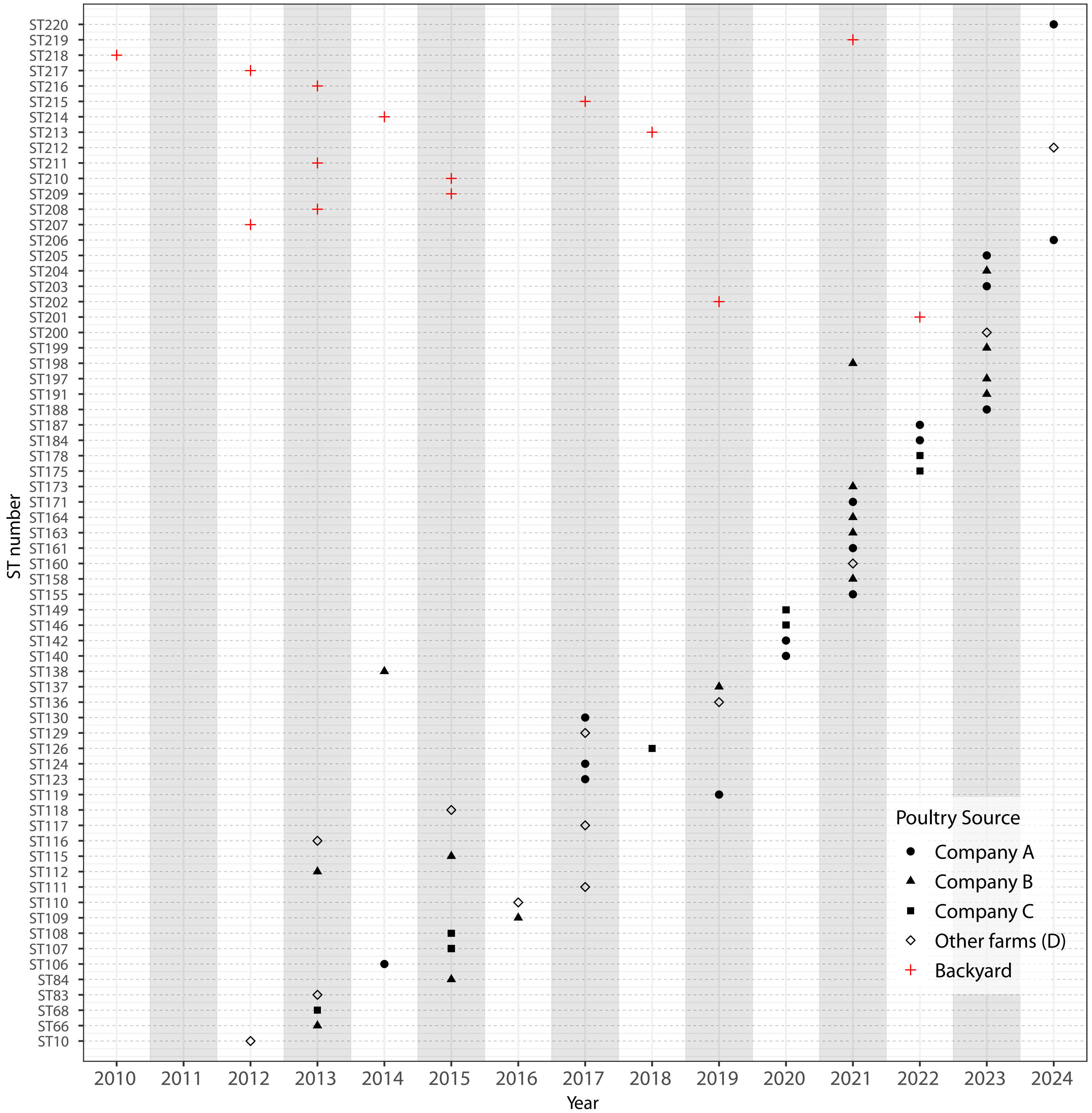

Figure 2S−A. *adk* allele frequencies (%) in China (n = 181), Italy (n = 227), and the USA (n = 65).
Bars represent relative frequencies of each allele per country.
Red rectangular borders indicate unique (private) alleles to a single country.

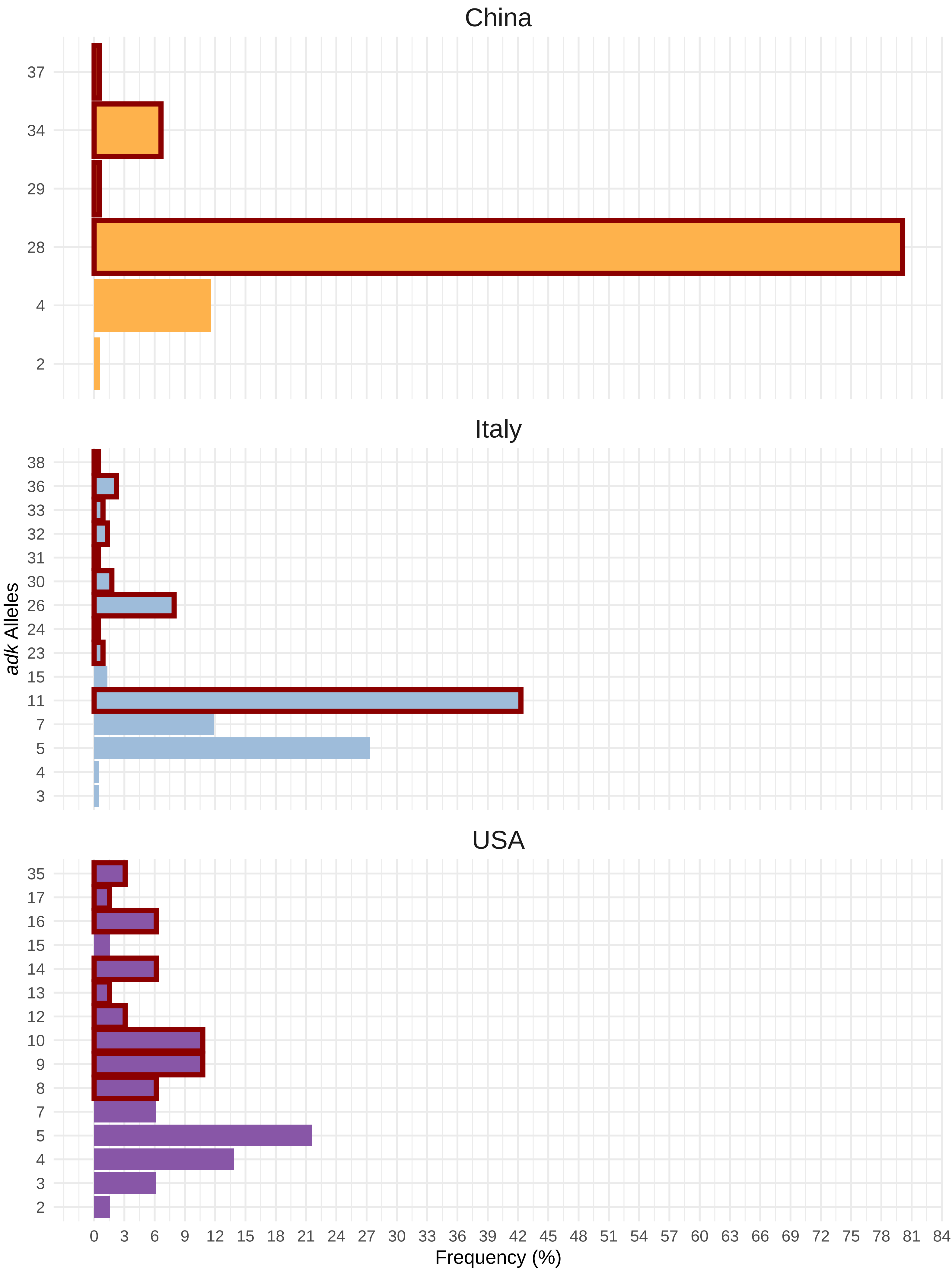

Figure 2S−B. *atpG* allele frequencies (%) in China (n = 181), Italy (n = 227), and the USA (n = 65).
Bars represent relative frequencies of each allele per country.
Red rectangular borders indicate unique (private) alleles to a single country.

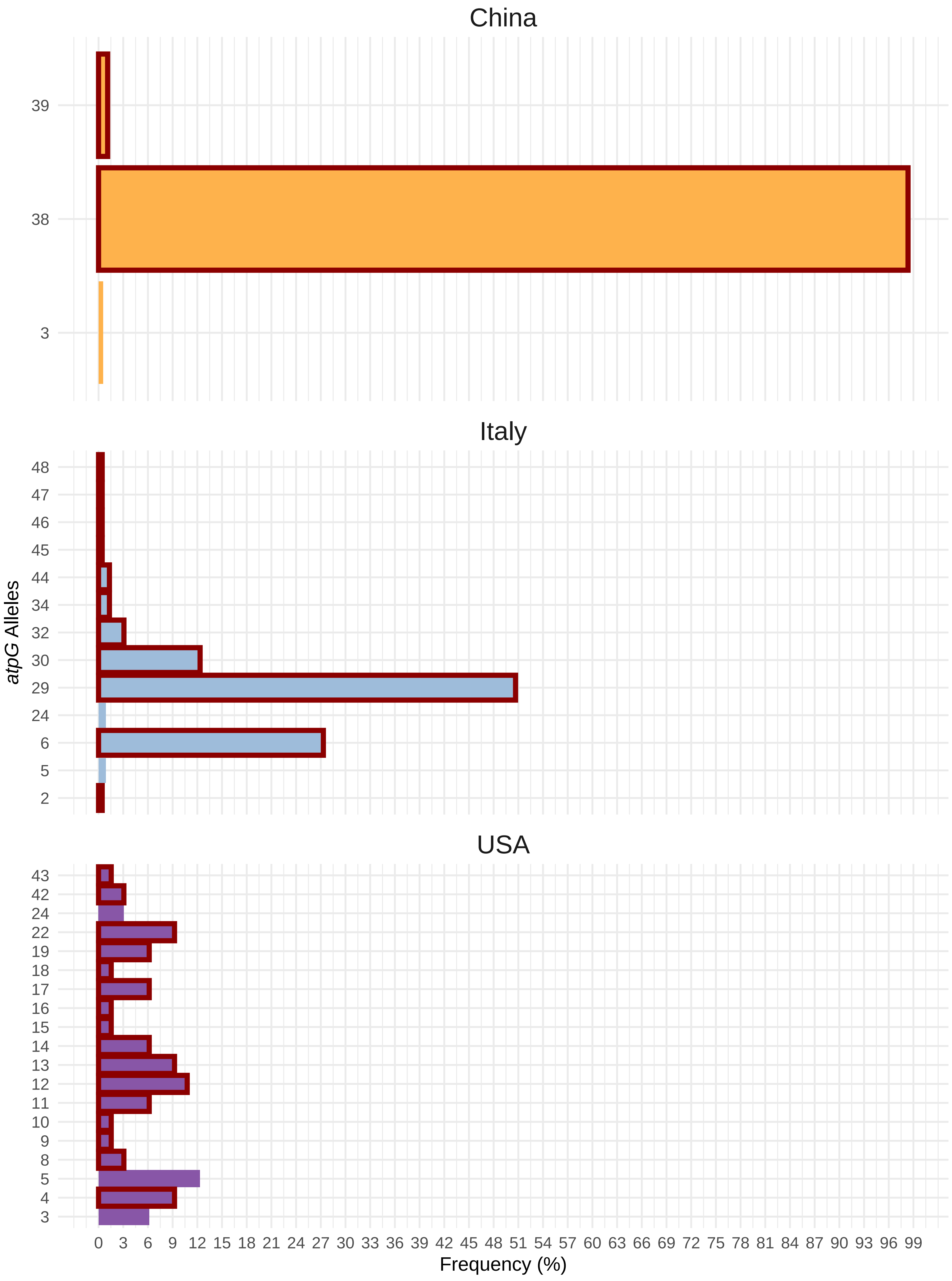

Figure 2S−C. *efp* allele frequencies (%) in China (n = 181), Italy (n = 227), and the USA (n = 65).
Bars represent relative frequencies of each allele per country.
Red rectangular borders indicate unique (private) alleles to a single country.

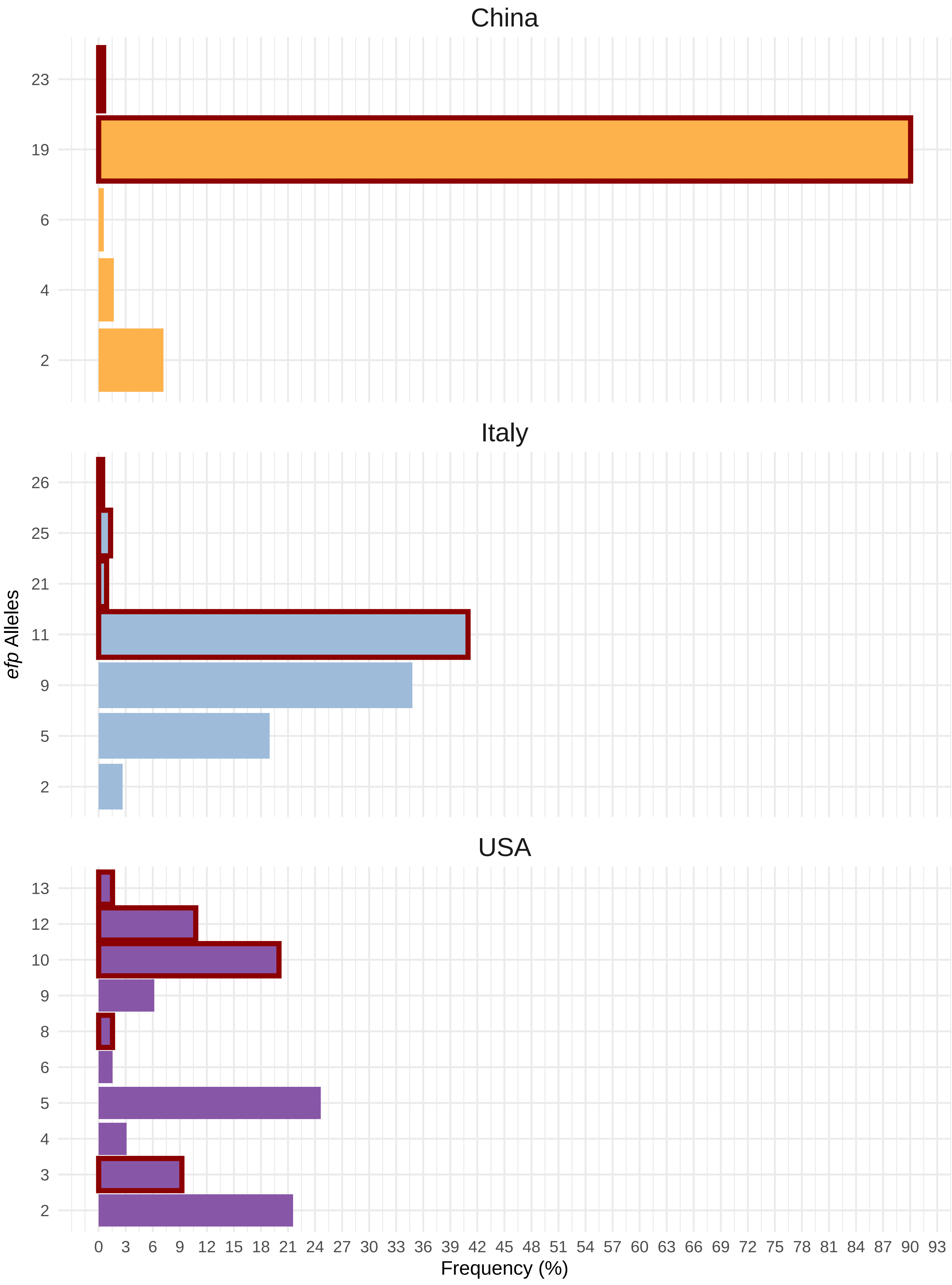

Figure 2S−D. *gmk* allele frequencies (%) in China (n = 181), China (n = 227), and the USA (n = 65).
Bars represent relative frequencies of each allele per country.
Red rectangular borders indicate unique (private) alleles to a single country.

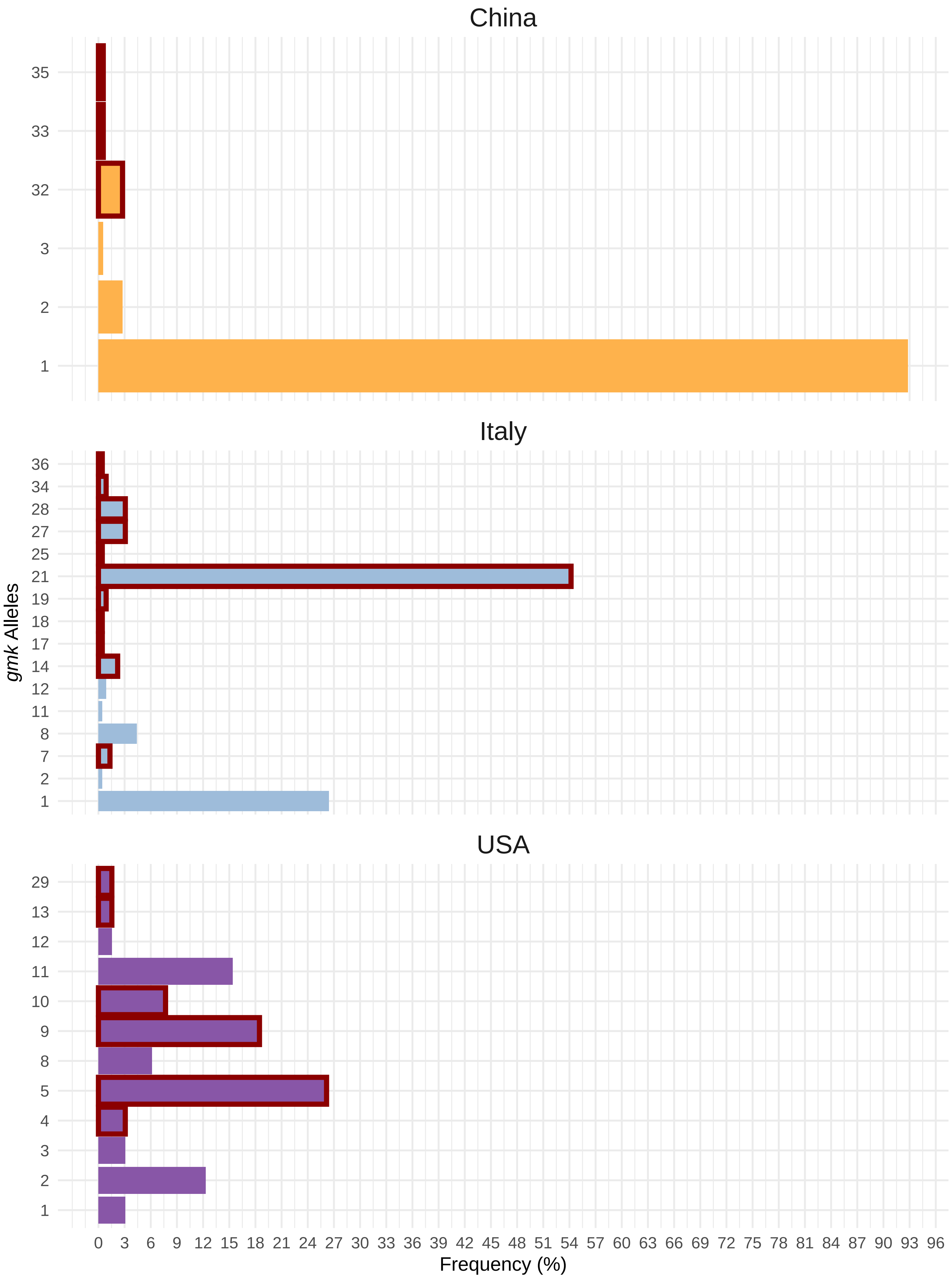

Figure 2S−E. *nagC* allele frequencies (%) in China (n = 181), Italy (n = 227), and the USA (n = 65).
Bars represent relative frequencies of each allele per country.
Red rectangular borders indicate unique (private) alleles to a single country.

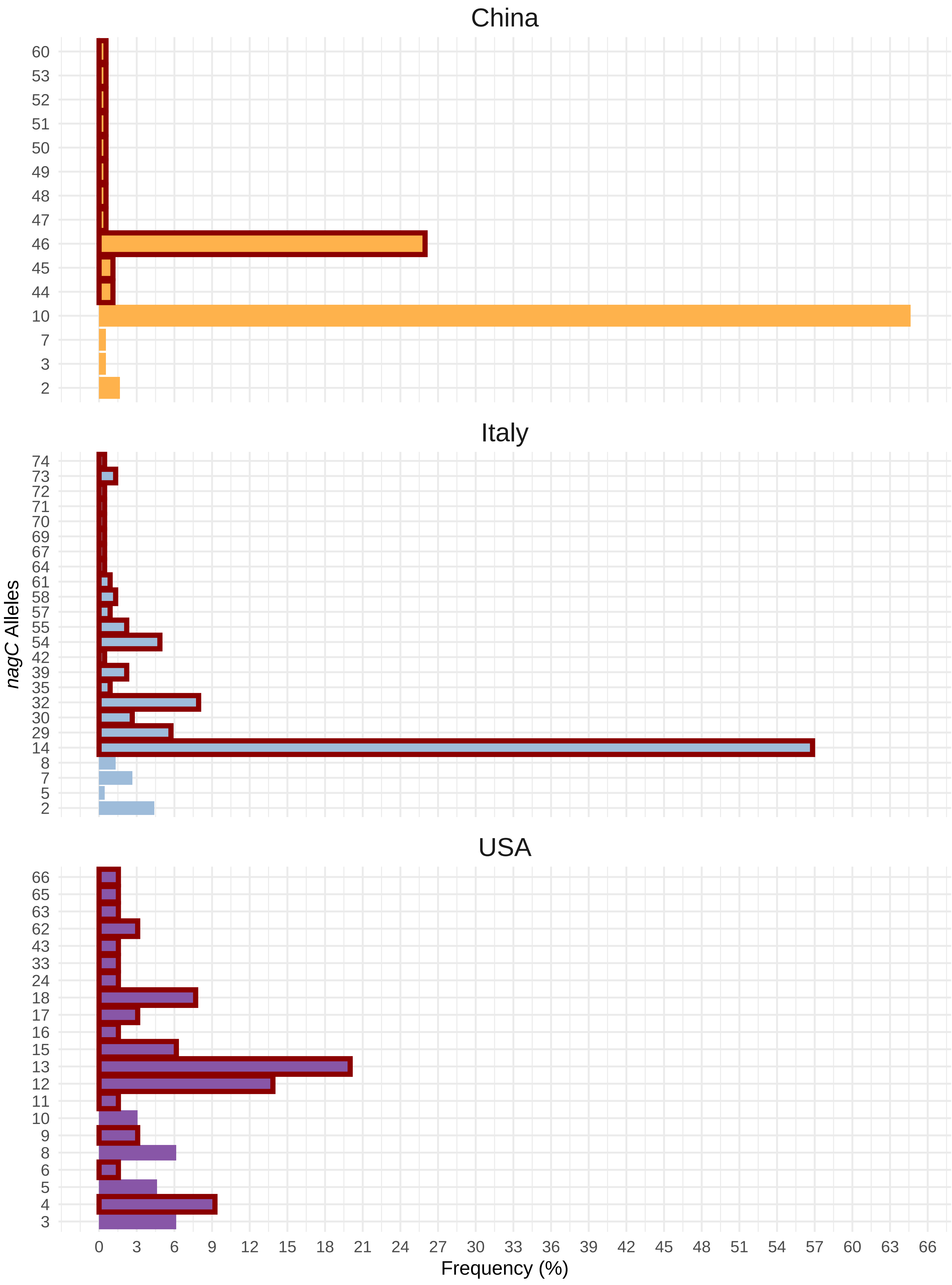

Figure 2S−F. *ppa* allele frequencies (%) in China (n = 181), China (n = 227), and the USA (n = 65).
Bars represent relative frequencies of each allele per country.
Red rectangular borders indicate unique (private) alleles to a single country.

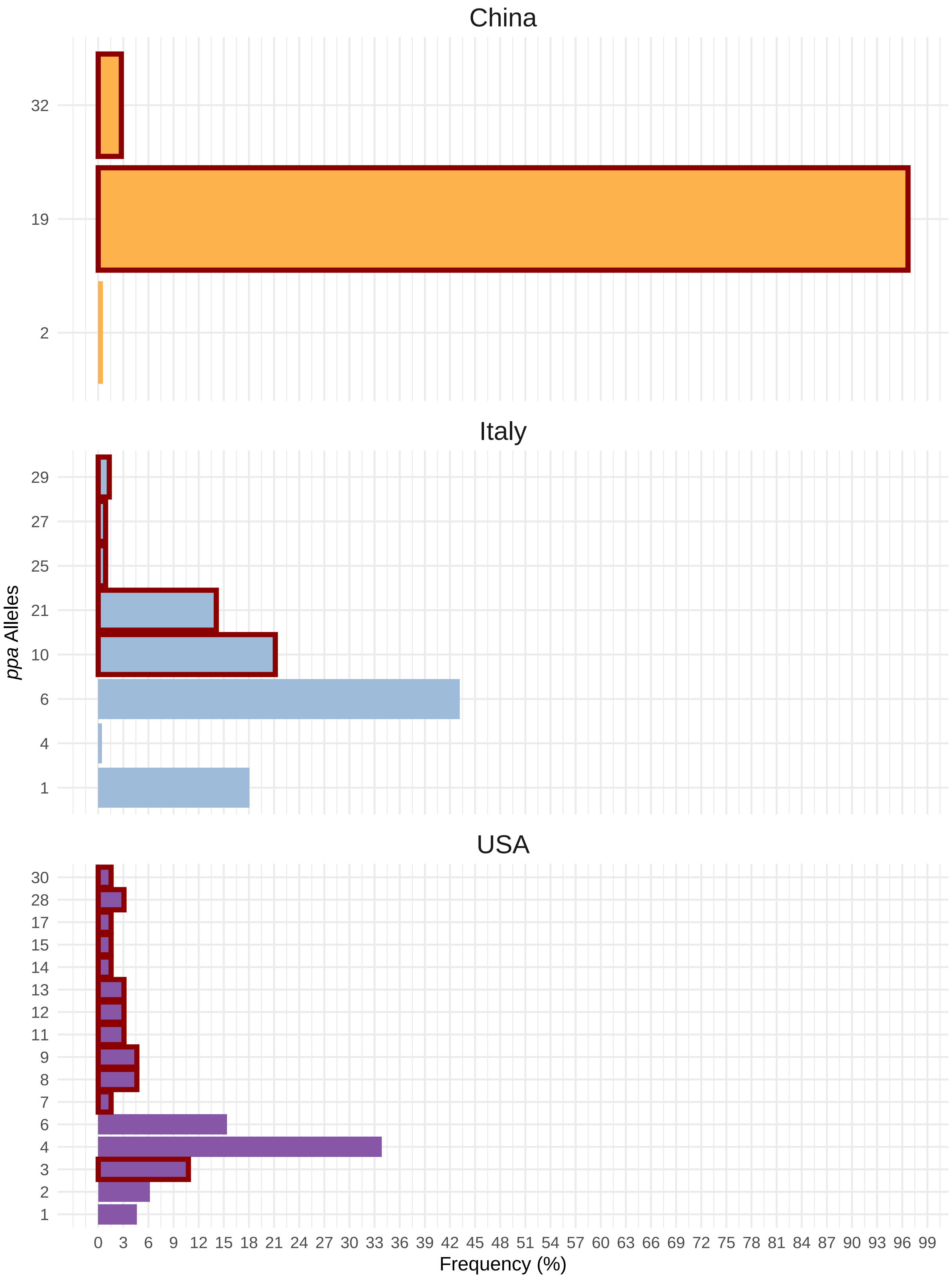

Figure 2S−G. *recA* allele frequencies (%) in China (n = 181), China (n = 227), and the USA (n = 65).
Bars represent relative frequencies of each allele per country.
Red rectangular borders indicate unique (private) alleles to a single country.

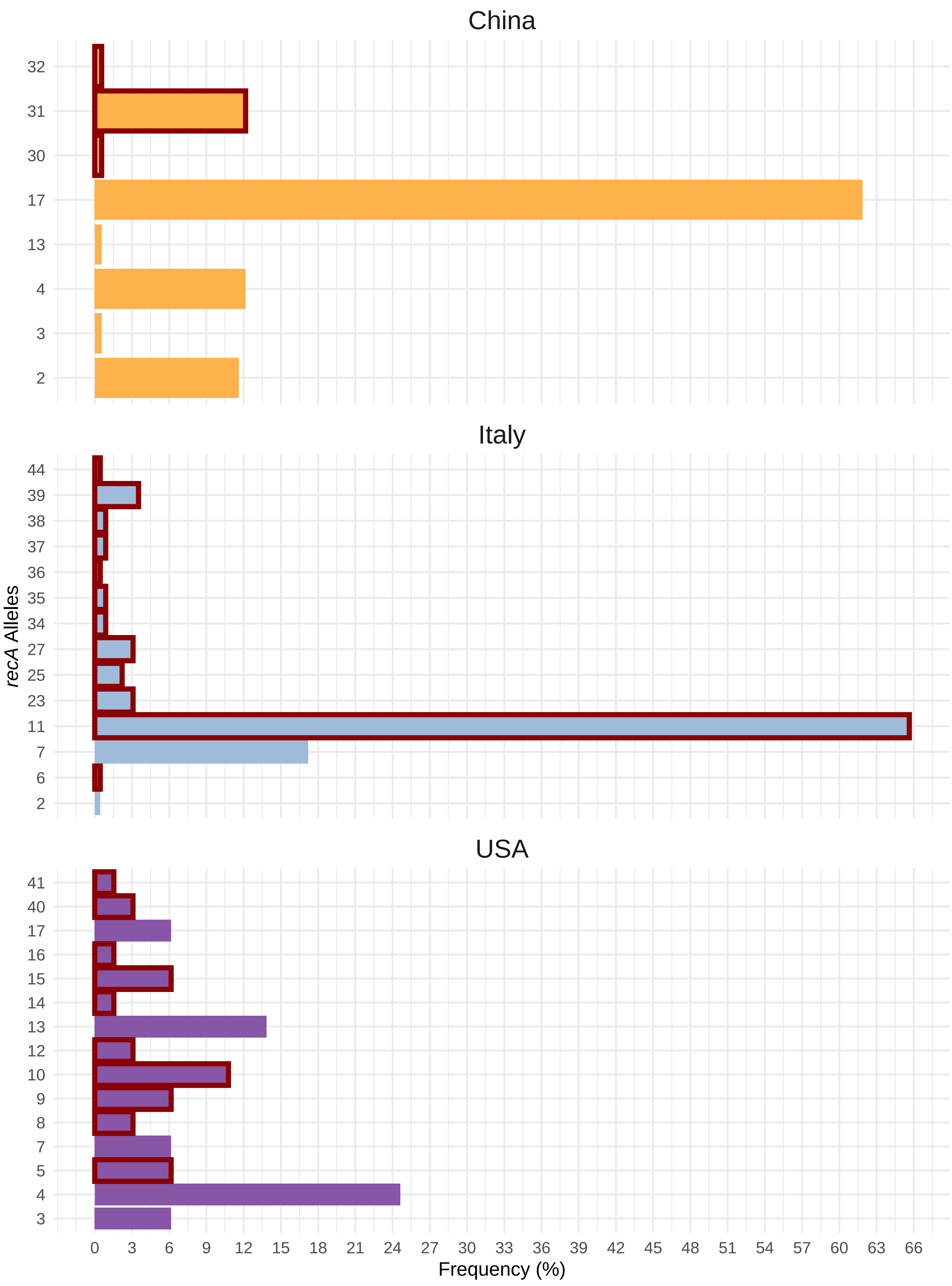

Supplement: Supplemental figures — Fig. S1 and S2. [file aem.01324-25-s0001.pdf]
