# Supplementary figures and images for: Molecular typing of Mycoplasma synoviae in industrial and backyard poultry: a 14-year study in Italy
Source: Appl Environ Microbiol. 2026 Mar 12;92(4):e01324-25. doi: 10.1128/aem.01324-25 (PMC13101485; doi:10.1128/aem.01324-25)

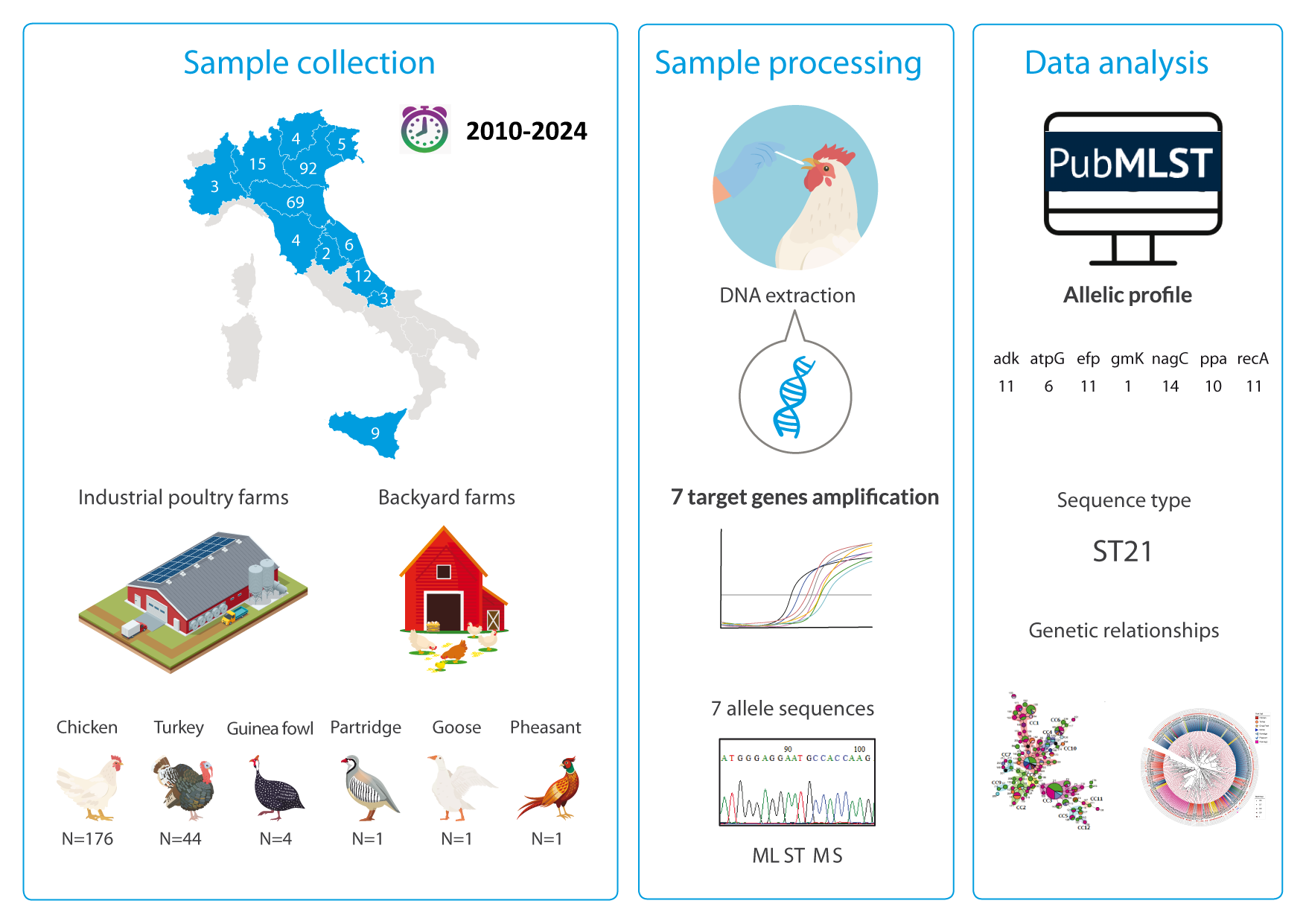

Supplement: Graphical abstract — Visual depiction of the study. [file aem.01324-25-s0002.tif]
